# Examining the impact of perceived psychological distances of quitting and continuing tobacco smoking on antismoking intention: a cross-sectional study

**DOI:** 10.1038/s41598-023-50440-6

**Published:** 2023-12-27

**Authors:** Domonkos File, Beáta Bőthe, Zsolt Demetrovics

**Affiliations:** 1 Institute of Psychology, ELTE Eötvös Loránd University, Budapest, Hungary; 2 Département de Psychologie, Université de Montréal, Montreal, Canada; 3 Centre of Excellence in Responsible Gaming, University of Gibraltar, Gibraltar, Gibraltar

**Keywords:** Psychology, Health care, Public health

## Abstract

To date, little research has been conducted to understand the role of psychological distances on smoking behaviour. Construal Level Theory posits that individuals mentally construe events, objects, or ideas based on their perceived distance in terms of spatial, temporal, social, and hypothetical dimensions, influencing their judgments and decision-making processes. The aim of the current study was (1) to provide a comprehensive exploration of psychological distances of costs and benefits of tobacco smoking and antismoking intention and (2) to examine whether smoking can be attributed to rational behaviour based on the psychological distance weighted balance of perceived costs and benefits of quitting and continuing smoking. Mediation models delineating the relationships among temporal and hypothetical psychological distances, personal relevance and antismoking intention were tested on cross-sectional survey data of 1486 smokers (880 men, *M*_*ag*e_ = 39.9 years, SD = 13.36). Psychological distances were shown to be important factors in the cognitive evaluation process of smoking behaviour. Perceived temporal distance to smoking continuation/cessation was related to personal importance and hypothetical psychological distances, which were associated with anti-smoking intention. Furthermore, antismoking intention was related to the psychological distance-weighted gain-cost balance of quitting and continuing smoking. The current findings enhance our knowledge of the cognitive evaluation of the outcomes of smoking, indicating that the choice of not quitting smoking may be partially based on a biased rational decision-making process.

## Introduction

Tobacco smoking is a major public health problem worldwide, being the leading cause of preventable death and disease^1^. Although substantial scientific evidence has been published on the implications of tobacco use for morbidity and mortality in the past 70 years^2^, the prevalence of smoking remained high; about 19% of the adult population in the US^3^ and 29% in Europe^4^ were current users. Although the cognitive and psychological underpinnings of smoking have been the subject of extensive research, there is a notable need for enhanced comprehension regarding the evaluation of smoking consequences, including their impact on both cessation and continued smoking, as well as their connection to the intention to quit.

The Construal Level Theory, which addresses the psychological distance (PD) that people subjectively experience about a future event^5,6^, has the potential to provide a better understanding of the psychological reasons for underestimating the costs of the seemingly irrational habit of tobacco smoking^7^. Construal Level Theory proposes that people perceive PDs towards specific events^5,6^, and the perceived distance correlates with behavioural intentions^8,9^. PD is a construct of four dimensions: temporal, hypothetical, spatial, and social distances^10^. Temporal distance refers to the perceived difference in time between the present moment and a future or past event. In contrast, hypothetical distance refers to the perceived level of uncertainty or likelihood associated with a particular event or scenario. Spatial distance refers to the perceived physical or geographic separation between an individual and an event, object, or location. Social distance pertains to the perceived level of closeness or similarity between an individual and others in a social context^10^. Each dimension has one end perceived close to the self and the other far away from it. As the perceived distance increases, the intensity of the affective response decreases^11^. As a consequence, the behavioural modification effect of psychologically distant events is smaller than that of proximal effects, as shown by studies on climate change^9,12^, economic behaviour^13^ or morality^14^. To date, little research has been conducted to understand the role of perceived PDs on smoking behaviour. Prior studies focused on the possibility of altering temporal distances through the temporal framing of messages communicating the health-related effects of smoking, with varying results. Kim and Kim^7^ reported that participants exposed to a near-future framed antismoking message on health consequences of smoking reported shorter perceived temporal PD, greater personal relevance and hypothetical PD to the risk portrayed in the message, and greater intention to quit smoking than participants exposed to the distant-future frame. Zhao et al.^15^ and Nan et al.^16^ tested the effects of temporal framing on cognitive, attitudinal, and behavioural outcomes by manipulating temporal PDs of antismoking messages and reported no significant main effect of temporal framing on the evaluated effectiveness of the messages.

In the context of smoking, choosing inaction may potentially lead to a collection of uncertain but high-cost consequences that could manifest far in the future (e.g., health-related adverse effects). Conversely, taking action involves relatively lower costs (e.g., dealing with cravings), but with a higher likelihood of occurrence in the near future. Since the negative consequences of smoking are slow-moving, they cannot easily be experienced directly^17^, which leads to a statistical assessment of risks, often resulting in a low behavioural modulatory effect^18^. Smokers are particularly prone to discounting delayed effects, with a distortion towards health losses: delayed health losses are more steeply discounted than health gains^19^. Considering the temporal sensitivity of smokers towards future consequences, observing the balance of positive and negative consequences of smoking weighted by their perceived distances (both in time and probability) potentially contributes to a better understanding of behaviour from the perspective of rational addiction theory^20^. This approach models addictive behaviour as a rational, forward-looking behaviour, where the behaviour is motivated by the immediate payoff of consumption and regulated by the future effects of consumption on the individual^21^. According to its economic origins, the theory was mainly tested in the context of economic environment variables, such as socioeconomic status^22^, excise taxes^23,24^, price^25^ or smoking ban in public areas^23^. The present article argues that smoking and cessation consequences weighted by the corresponding PDs and subjective importance can successfully represent the uncertainty and variable information distribution^26^ present in the assessment of addiction-related scenarios. Thus, mapping the temporal and probabilistic characteristics of payoffs and future costs might contribute to a better understanding of the evaluation process underlying the addictive behaviour.

The current study, therefore, aims to provide a comprehensive exploration of PDs of costs and gains of tobacco smoking and quitting, examining temporal and hypothetical dimensions. Similarly to previous studies^7,15,16^, only temporal and hypothetical dimensions were examined, as spatial and social distances might have limited relevance to smoking. While health consequences in terms of social distances can be understood within the Construal Level Theory framework (i.e., effects of second-hand smoke), this is not applicable to other outcomes like recreation or weight control. The proposed approach differs notably from previous studies in PDs in that perceived distances were examined for both the scenario where individuals would maintain their current behaviour (inaction: continue smoking) or for the scenario where they would take action (action: stop smoking). The study aimed to investigate the effects of perceived PDs towards costs and gains of action and inaction on anti-smoking intention. Different from previous studies^7,15,16^, PDs of consequences beyond health-related effects were investigated, including gains (such as social facilitation, enhancement, stress management, rituals, weight control and performance) and costs (such as financial status, psychological wellbeing, desired lifestyle, social environment, fitness, appearance). Hypotheses were formulated based on the causal model proposed by Kim and Kim^7^. The original Kim and Kim model structure was employed, albeit with the adaptation of substituting measured variables of temporal and hypothetical PDs and personal relevance with latent variables. This adjustment was made due to the investigation of various smoking-related consequences. The subsequent hypotheses were constructed. It was expected that the perceived temporal distance was related both the perceived hypothetical distance (H1) and personal relevance (H2a); the closer a consequence was perceived, the greater the likelihood of its occurrence and its personal relevance was. Also, a positive relationship was assumed between personal relevance and hypothetical distance (H2b). Furthermore, we posited that perceived hypothetical distances (H3) and temporal distances mediated by hypothetical distances (H4) related to smoking-related consequences would relate to the intention to quit as follows: (1) perceived distances of positive consequences of quitting smoking, and (2) negative consequences of continuing smoking were expected to have a negative correlation with the intention to quit (i.e., the closer they were perceived, the greater the intention to quit was). However, in case of (3) negative consequences of quitting and (4) positive consequences of continuing smoking, the opposite correlation was expected. Furthermore, we hypothesized that personal relevance mediated by hypothetical distances would have a positive (in case of positive consequences of quitting smoking and negative consequences of continuing)/negative (in case of negative consequences of quitting smoking and positive consequences of continuing) effect on intention to quit (H5). A secondary aim of the study was to investigate whether smoking can be attributed to rational behaviour by observing the balance of costs and gains of action and inaction, weighted by the corresponding perceived temporal and hypothetical PDs and personal relevance. We hypothesized that the gain-cost balance would predict intention to quit smoking: higher gains relative to costs would be associated with a lower intention to quit (H6a), and the planned quit date would be further in the future (H6b), and the importance of quitting would be lower (H6c).

## Method

### Procedure and participants

Data were collected from popular Hungarian news portals using an online survey from July to August in 2022. The study was advertised as a research project concerning the psychological factors of tobacco smoking. Written informed consent was obtained from participants before data collection, and participants were ensured of their anonymity. The present study was conducted adhering to the Declaration of Helsinki and was approved by the institutional ethical review board of the Joint Committee of Ethics of the Psychology Institutes, Hungary (Number 2021/430). No personal information that allowed personal identification was asked, and a secure online platform (Qualtrics Research Suite; Qualtrics, Provo, UT) was used for data collection.

The inclusion criteria were (i) providing informed consent, being (ii) aged 18 years or older and (iii) current smoker. Out of the 3197 respondents who began the survey, 1488 (46.5%) did not finish it. Additionally, 223 (6.9%) identified themselves as non-smokers by selecting the response "I do not smoke" to the question "How often do you smoke?". Consequently, these individuals were excluded from all subsequent analyses. Overall, 1486 participants (880 men, 59.2%; 601 women, 40.4%; and 5 selected prefer not to respond option, less than 0.5%) aged between 18 and 84 years (*M*_*ag*e_ = 39.9 years, SD = 13.36) completed the survey. Of these, less than 1% had maximum primary education, 13.7% reported having a vocational degree, a further 17.4% had high school degree, and 67.1% had college or university degree. Regarding relationship status, 24.3% were single, 74% were in any kind of romantic relationship (i.e., being in a romantic relationship or married) and 1.7% chose the “other” option. A total of 1018 respondents indicated regular use of packaged cigarettes, 344 reported using rolling tobacco, 428 mentioned using electronic cigarettes, 64 stated they used cigars, and 25 subjects reported regular use of a pipe. It is important to note that the total number exceeds 1486, as respondents could select more than one answer option.

### Measures

**Demographic status.** Standard demographic questions such as gender, age (in years), education, and relationship status were asked, see Appendix 2.

**Types of tobacco products.** The assessment of tobacco product types was conducted using a single-item measure, asking participants, “Which tobacco product do you use on a regular basis?” The options provided were (1) packed cigarettes, (2) rolling tobacco, (3) electronic cigarettes, (4) cigars, and (5) pipe, allowing for multiple selections.

**Nicotine dependence** was measured with the Fagerström Test for Nicotine Dependence^27^. It contains six items to assess the quantity of cigarette consumption, the compulsion to use, and dependence. Scores range from 0 to 10, the higher the total score, the more intense the patient’s physical dependence on nicotine is.

**Anti-smoking intention**, encompassing the intention to quit smoking, planned date, and perceived importance of quitting, were assessed using three single-item measures. Using single-item measures was based on the study of Hummel et al.^28^, which indicated that a single-item measure of quitting intention outperformed in predictive validity of quit attempts both the Stages of Change measure and the Motivation to Stop Scale. Given the extensive nature of the questionnaire, we opted for this solution to reduce the number of items. Notably, an adjustment was made to the original “Are you planning to quit smoking within the next 6 months?” item, dividing it into two items to obtain a more nuanced understanding: (1) intention to quit (“Do you ever plan to quit smoking?” [5-point scale, 1-certainly not to 5-certainly yes]) and (2) the planned date of quitting in accordance with the transtheoretical model (“When do you plan to quit smoking?” [1-within a week; 2-within a month; 3-within 6 months; 4-within 12 months; 5-within 5 years; 6-within 10 years; 7-over 10 years; 8-don’t plan]). Additionally, we introduced another item on the perceived importance of quitting, "How important is it for you to quit smoking?" (5-point scale, 1-not at all to 5-extremely), closely resembling the one used by Kahler et al.^29^, given that the desire to quit smoking has been identified as a crucial predictor for successful smoking cessation^30^. In the structural models, the measured variables were introduced individually to represent the variables of Intention to quit, Planned date of quitting, and Importance of quitting.

**Past smoking behavior and regret** about starting smoking were assessed using single-item measures. Participants were asked one question assessing past quitting attempts: Have you tried to quit smoking in the past 12 months? (*yes, no*). One item evaluated the feeling of regret regarding smoking, using the following statement: “I wish I had never started smoking.” (5-point scale, 1-*totally disagree*, 5-*totally agree*). The reason for including the assessment of regret stems from prior research^31,32^ which indicated that regret could be a pivotal factor in understanding the experiences of smokers who persist in smoking despite their desire to quit. These variables were not included in the structural models to minimize deviations from the original Kim and Kim^7^ model. The reason for assessing these variables was to offer additional context and insights that could enhance the understanding and interpretation of the outcomes obtained within the study.

**Psychological distances** were measured along two dimensions: hypothetical and temporal distances. Both dimensions were assessed for action (i.e., if the respondent stops smoking) and inaction (i.e., if the respondent does not stop smoking). Items were based on literature presenting perceived consequences of smoking, reasons for quitting smoking, and motivation to smoke^33–37^. Fourteen consequences were identified: social facilitation, health, enhancement, financial status, performance, desired lifestyle, psychological wellbeing, social environment, rituals, stress management, craving, fitness, appearance, weight control. Each consequence was assessed for hypothetical and temporal distances for action and inaction, using one item (see Appendix 1). Answers were indicated on a 6-point Likert scale for hypothetical distances (1-*very likely* to 6-*very unlikely*). Answers were indicated on an 11-point Likert scale for temporal distances. Based on Herd, Borland and Hyland^38^ work, time differences between answer choices increased with a logarithmic function. In case of action, answer options ranged from “immediately” to “later than 5 years”, while in case of inaction, they ranged from “immediately” to “later than 20 years”.

### Personal relevance of smoking consequences

Personal relevance of each of the investigated 14 consequences (7 positive, 7 negative) were assessed with the question: “Please indicate how important the following aspects of smoking are to you.”. Answers were indicated on a 5-point Likert scale (1-*not at all*, 5-*very important*). For the items see Appendix 3.

### Analyses

**Figure 1:**
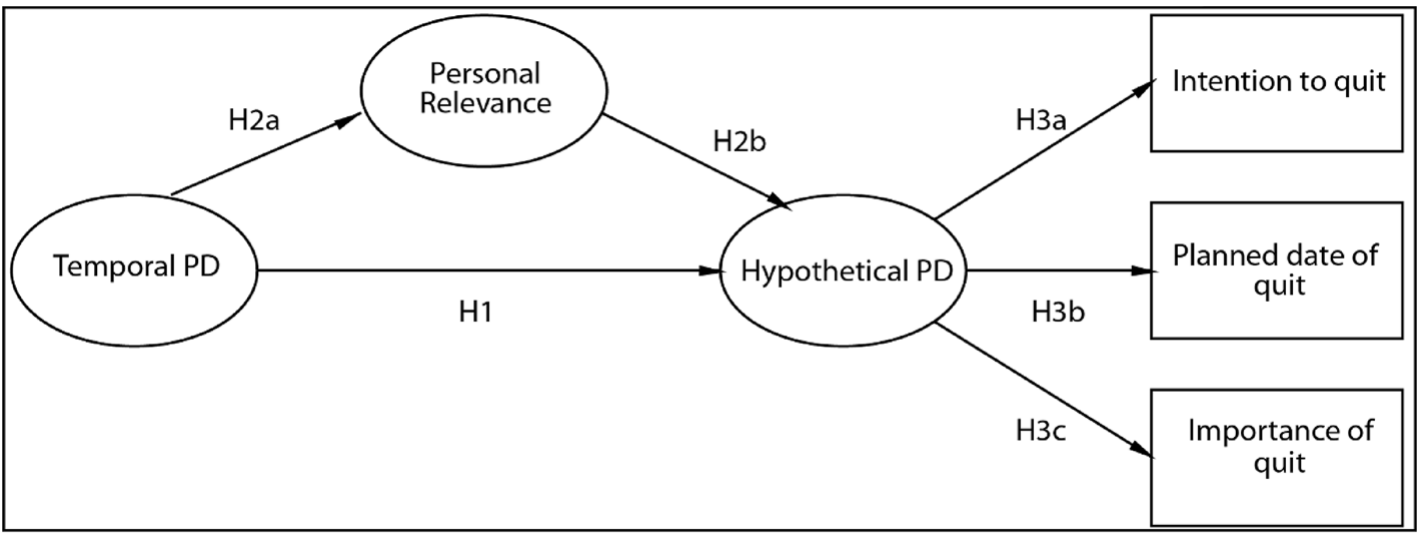
Graphical representation of the examined models.

Structural equation modelling (SEM) with latent variables were conducted to examine the relationships of PDs with personal relevance and anti-smoking intention (intention to quit smoking, its planned date, and perceived importance). The proposed model was based on the work of Kim and Kim^7^; for a graphical representation, see Fig. 1. This model was applied to two theoretical scenarios (quitting smoking [action]/continuing smoking [inaction]) for both the positive (gain) and negative (cost) consequences of smoking. Thus four models were investigated: Inaction-cost, Inaction-gain, Action-cost and Action-gain. The Inaction-cost Model delved into the hypothetical scenario where smokers would not change their current smoking behaviour (i.e., continue smoking) and assessed the psychological distances and personal relevance related to the adverse outcomes of this behaviour (i.e., the likelihood and timing of specific consequences) and their influence on the intention to quit smoking. In this model, the latent variable of Temporal and Hypothetical PD was constructed from the time and likelihood estimations of the negative consequences of continued smoking, while latent variable Personal Relevance was constructed from the personal relevance scores of the consequences included in the PD latent variables. In the Inaction-gain Model, the examination focused on the theoretical situation where smokers also maintain their current smoking behaviour (i.e., continue smoking). This model evaluated the psychological distances and individual significance associated with the positive consequences of this behaviour, encompassing the probability and timing of specific outcomes, and their impact on the intention to quit smoking. In this model, the latent variable of Temporal and Hypothetical PD was derived from assessments of time and likelihood regarding the positive consequences of ongoing smoking. Additionally, the latent variable of Personal Relevance was constructed from the significance scores attributed to the consequences incorporated within the PD latent variables. Furthermore, in the Action-gain Model, the hypothetical scenario where smokers would terminate their current smoking behaviour (i.e., quit smoking) was tested. Psychological distances and personal relevance related to the positive outcomes of quitting (i.e., the likelihood and timing of specific consequences) and their influence on the intention to quit smoking were investigated. In this model, the latent variable of Temporal and Hypothetical PD was derived from assessments of time and likelihood regarding the positive consequences of quitting smoking. Additionally, the latent variable of Personal Relevance was constructed from the significance scores attributed to the consequences incorporated within the PD latent variables. Finally, in the Action-cost Model, the evaluation of the negative consequences of quitting smoking was investigated. In this model, the latent variable of Temporal and Hypothetical PD was derived from assessments of time and likelihood regarding the negative consequences of quitting smoking. Additionally, the latent variable of Personal Relevance was constructed from the significance scores attributed to the consequences incorporated within the PD latent variables. Considering the exploratory nature of the study, a liberal cut-off for factor loadings was used in the measurement models. Following the recommendations of Hair et al.^39^, measured variables were not included in the measurement model, if the standardized factor loadings were below 0.3 (for the structural and measurement models see Fig. 1).

To test the applicability of Rational Theory of Addiction on PDs, path analysis was conducted to examine the relationship between the difference of the summed PDs of the relevance weighted positive and negative consequences (Σ(gain_temporalPD_i_ × gain_hypotheticalPD_i_ × gain_relevance_i_) – Σ(cost_temporalPD_i_ × cost_hypotheticalPD_i_ × cost_relevance_i_)) on the intention to quit smoking, its planned time, and perceived importance (Model Rational). To test the contribution of the involvement of PDs in the equation, a control model (Model Rational-control) was formed to examine the relationship between the difference of the summed relevance weighted positive and negative consequences (Σ(gain_relevance_i_) − Σ(cost_relevance_i_)) on antismoking behaviour. All analyses were performed in R (4.0.2), package Lavaan^40^, with diagonally weighted least squares estimation. Distribution plots were made with package ggplot2^41^ and package ggridges^42^.

When assessing the models, multiple goodness of fit indices were observed^43^ with good or acceptable values based on the following thresholds^44,45^. Regarding the comparative fit index (CFI) and Tucker–Lewis index (TLI), values higher than 0.95 indicated that a model had good fit, whereas values higher than 0.90 indicated that a model had acceptable fit. Regarding the root mean square error of approximation (RMSEA) with its 90% confidence interval (90% CI), a model can be considered good if its RMSEA value is below 0.06, whereas it can be considered acceptable if this value is below 0.08. In addition, following Schellenberg et al.^46^ suggestions, to examine the significance of indirect pathways in the mediation model, 95% bias-corrected bootstrapped confidence intervals (CIs) with 5000 resample were computed.

The normality of the dependent variables was examined and did not violate the thresholds of Kim^47^, ⁠neither for skewness (ranging from − 0.70 to 0.32), nor for kurtosis (ranging from − 0.844 to − 0.13).

## Results

The average score of the sample for the Fagerström Test for Nicotine Dependence was 4.16 (SD = 2.85). One hundred and forty-two (9.5%) respondents did not plan to quit smoking, 156 (10.5%) was undecided, and 1188 (79.9%) was planning to quit smoke. One hundred and forty-one (9.5%) respondents planned to quit smoke within a week, 147 (9.9%) within a month, 265 (17.8) within six months, 266 (17.9%) within 12 months, 335 (23.9%) within five years, 94 (6.3%) within 10 years, 22 (1.4%) over 10 years, and 216 (14.5%) did not plan. One hundred and eighty-five (12.4%) respondents said that quitting smoking was not important, 374 (25.2%) were not sure, and 927 (62.4%) respondents said that quitting was important. Nine hundred and sixteen (71.2%) respondents regret that they had started smoking, 96 (7.5%) was undecided and 275 (21.3%) did not regret.

**Figure 2:**
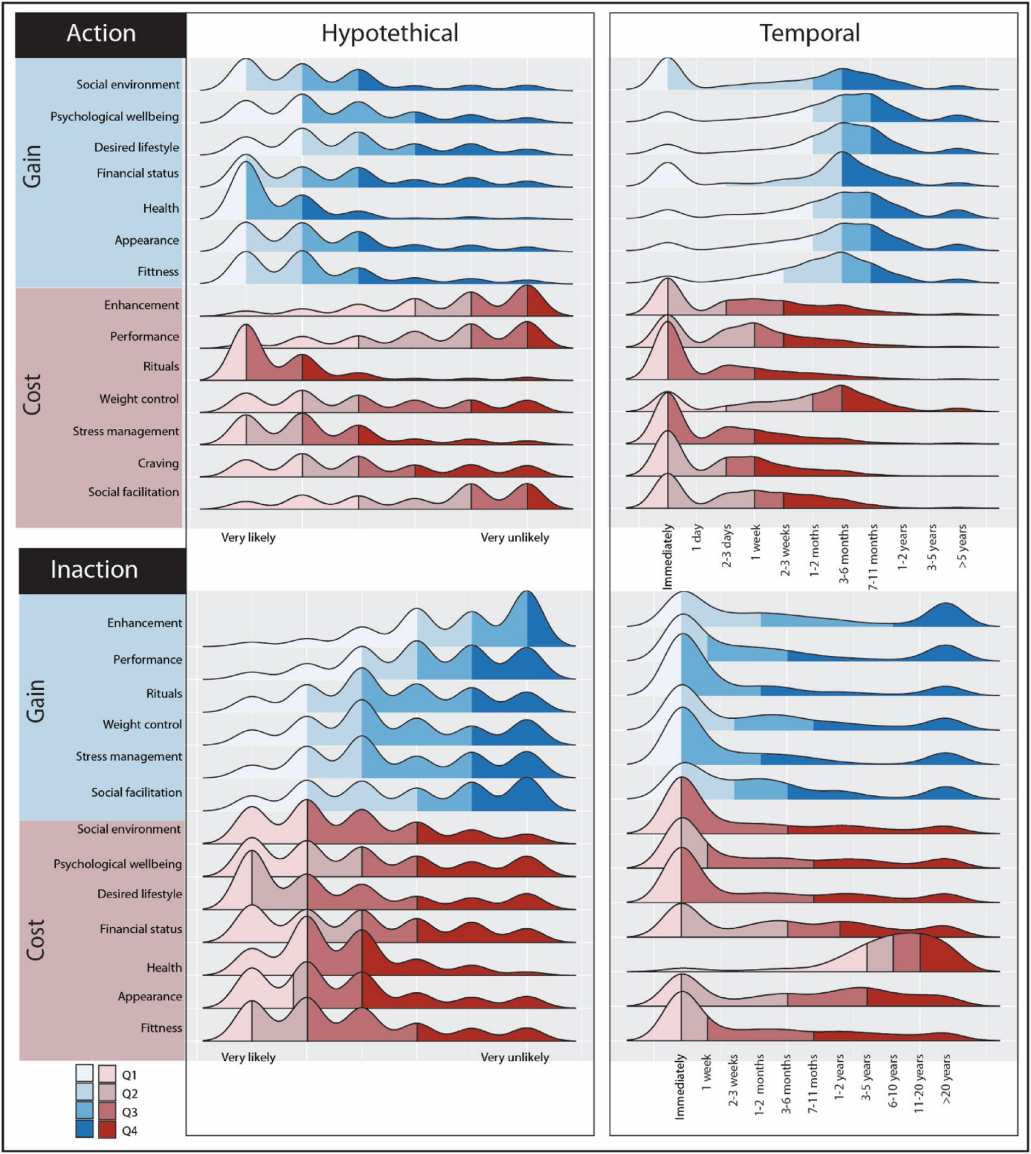
Distribution plots of psychological distances of action and inaction.

**Figure 3:**
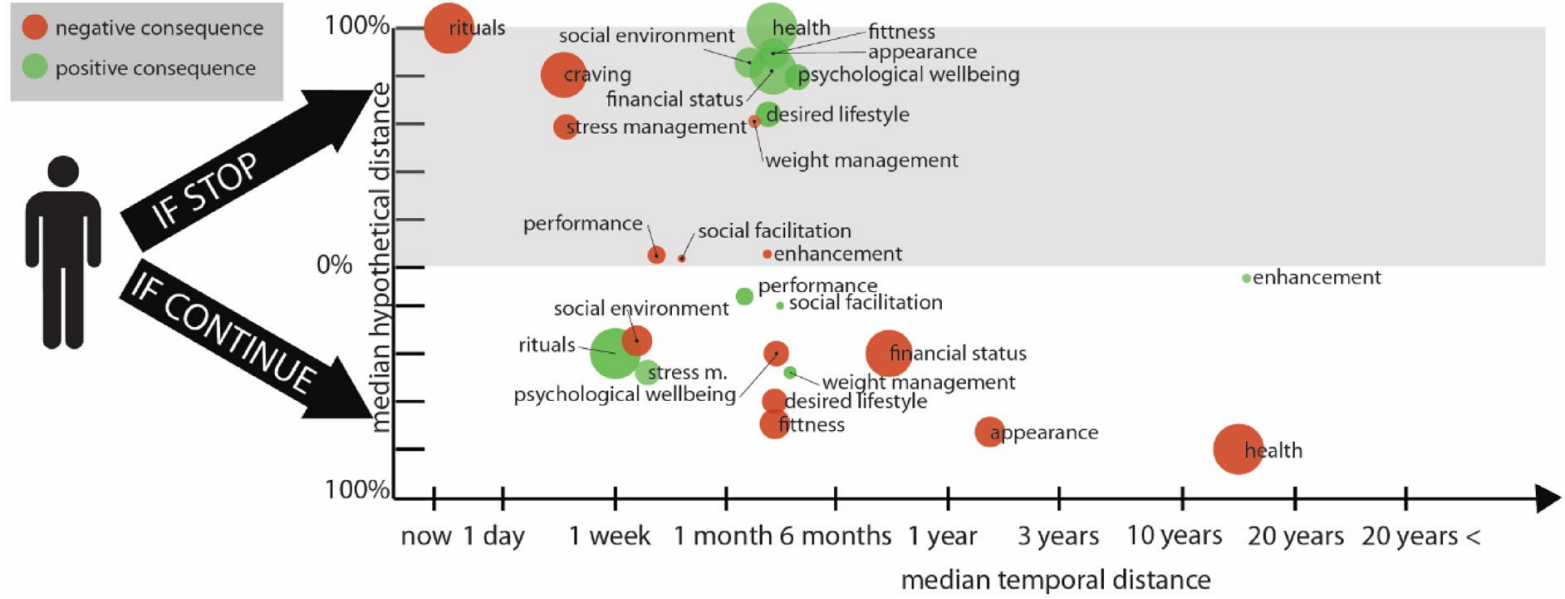
Visualization of perceived hypothetical and temporal distances pertaining to the positive and negative consequences of both persisting in and ceasing smoking. Each consequence is denoted by a disk, with red denoting negative outcomes and green denoting positive ones. The vertical position of the consequences indicates the median response for hypothetical distance, while the horizontal position represents the median response for temporal distance. The size of the disks corresponds to the level of personal relevance assigned to each specific consequence, with larger disks indicating greater personal significance.

Psychological distances of action and inaction-related consequences are presented in Table 1, and their distributions are visualized in Figs. 2 and 3. Perceived personal relevance of smoking-related costs and gains are reported in in Table 2. Correlations between the variables included in the SEM analyses are reported in Table 3.

**Table 1:** Mean and median hypothetical and temporal PDs for action and inaction, SD in parenthesis.

	Action				Inaction			
	Temporal PD		Hypothetical PD		Temporal PD		Hypothetical PD	
	Mean (SD)	Median	Mean (SD)	Median	Mean (SD)	Median	Mean (SD)	Median
Social facilitation	5.79 (3.58)	5	4.76 (2.1)	6	5.86 (3.85)	5	4.28 (2.11)	5
Health	6.95 (2.65)	7	1.73 (1.28)	1	8.91 (2.1)	10	2.77 (1.52)	2
Rituals	2.83 (2.56)	1	1.79 (1.38)	1	4.58 (4.05)	2	3.94 (1.94)	3
Financial status	6.09 (3.15)	7	2.82 (1.94)	2	5.84 (3.69)	6	3.34 (2.03)	3
Performance	5.89 (3.6)	5	4.99 (1.93)	6	5.71 (4.12)	5	4.73 (1.83)	5
Desired lifestyle	6.97 (2.55)	7	3.17 (1.87)	3	4.87 (3.97)	4	2.83 (2.06)	2
Psychological wellbeing	7.03 (2.64)	7	2.94 (1.82)	2	5.16 (3.97)	4	3.33 (2.12)	3
Social environment	5.39 (3.37)	6	2.55 (1.75)	2	4.45 (3.85)	3	3.04 (1.87)	2
Enhancement	6.44 (3.69)	7	5.21 (1.87)	6	7.1 (3.88)	10	5.41 (1.68)	6
Stress management	4.08 (3.26)	3	3.5 (2.05)	3	5.01 (4.12)	3	4.05 (2.01)	3
Fitness	3.13 (2.66)	3	2.51 (1.66)	2	4.94 (3.86)	4	3.01 (1.93)	2
Appearance	6.57 (2.3)	7	2.43 (1.65)	2	6.06 (3.66)	7	2.9 (1.86)	2
Craving	6.96 (2.57)	7	2.63 (1.74)	2				
Weight management	6.29 (3.12)	7	3.53 (2.12)	3	5.55 (3.96)	5	3.95 (1.98)	3

Answers were indicated on a 6 point Likert scale for hypothetical distances (1-very likely to 6-very unlikely). Answers were indicated on a 11-point Likert scale for temporal distances (1-immediately to 11-for action: > 5years, for inaction: > 20 years).

**Table 2:** Personal relevance of smoking-related factors, SD in parenthesis.

Health	3.67 (1.25)
Financial status	3.53 (1.24)
Desired lifestyle	2.99 (1.30)
Psychological wellbeing	3.02 (1.39)
Social environment	3.19 (1.23)
Fitness	3.27 (1.26)
Appearance	3.17 (1.25)
Craving	3.43 (1.27)
Social facilitation	2.51 (1.20)
Rituals	3.61 (1.13)
Performance	2.89 (1.24)
Enhancement	2.56 (1.17)
Stress management	3.31 (1.23)
Weight control	2.65 (1.28)

Answers were indicated on a 5-point Likert scale (1-not at all, 5-very important).

**Table 3:** Pearson correlation coefficients between latent and exogenous variables used in the structural equation models.

	1	2	3	4	5	6	7	8	9	10	11
1 Action-gain hypothetical PD	1.0										
2 Action-gain temporal PD	− 0.69*	1.0									
3 Gain personal relevance	0.75*	− 0.52*	1.0								
4 Inaction-cost hypothetical PD	0.74*	− 0.47*	0.79*	1.0							
5 Inaction-cost temporal PD	− 0.57*	0.41*	− 0.73*	− 0.78*	1.0						
6 Inaction-gain hypothetical PD	− 0.04	0.04	− 0.08*	− 0.03	− 0.05	1.0					
7 Inaction-gain temporal PD	− 0.06*	0.03	− 0.08*	− 0.07*	0.31*	− 0.80*	1.0				
8 Cost personal relevance	0.03	0.01	0.02	0.05	− 0.16*	0.89*	− 0.83*	1.0			
9 Intention to quit	0.55*	− 0.34*	0.50*	0.56*	− 0.40*	− 0.18*	0.08*	− 0.11*	1.0		
10 Planned date of quitting	− 0.58*	0.39*	− 0.49*	− 0.55*	0.41*	0.15*	− 0.08*	0.09*	− 0.70*	1.0	
11 Importance of quitting	0.68*	− 0.40*	0.61*	0.69**	− 0.49*	− 0.16*	0.08*	− 0.10*	0.71*	− 0.70*	1.0

*p < 0.01.

**Figure 4:**
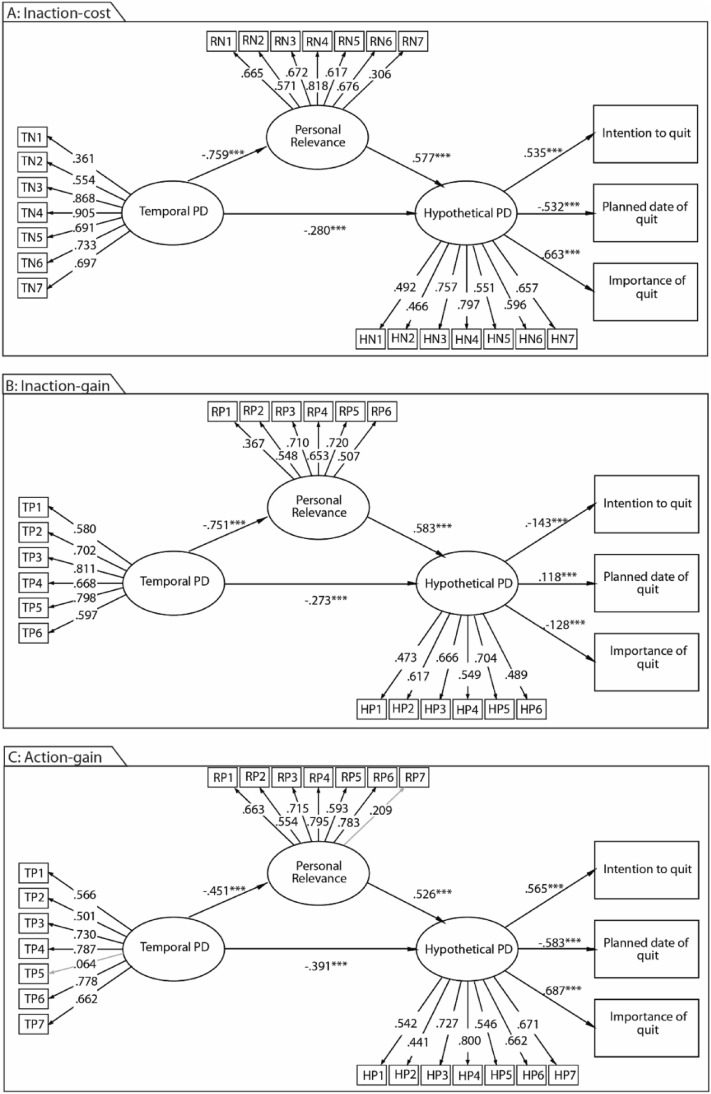
The structural equation modelling outcomes elucidate the relationship between temporal psychological distance, personal relevance, hypothetical psychological distance, and their impact on the intention to quit smoking, the scheduled quit date, and the perceived significance of the cessation effort. (**A**) Inaction-cost Model. (**B**) Inaction-gain Model. (**C**) Action-gain Model. The coefficients represent standardized factor loadings in the case of the measurement models and standardized regression weights when examining the associations between the variables.

The Inaction-cost Model showed an acceptable fit to the data; CFI of 0.963, TLI of 0.959 and RMSEA of 0.073 [90% CI 0.071–0.076]. The analyses showed (see Table 4A and Fig. 4A) that temporal PDs had a negative, moderate association with personal relevance (H2a, β = − 0.280 [95% CI − 0.336, − 0.224], *p* < 0.0001) and a negative, strong association with hypothetical PDs (H1, β = − 0.759 [95% CI − 0.779, − 0.738], *p* < 0.0001). The more distant the costs of smoking were perceived in the future, the lower the personal relevance and the hypothetical distance were. Temporal PDs mediated by hypothetical PDs had a negative and weak association with intention to quitting (H4a, β = − 0.150 [95% CI − 0.180, − 0.119], *p* < 0.0001) and perceived importance of quitting (H4c, β = − 0.186 [95% CI − 0.224, − 0.148], *p* < 0.0001) as well as had a positive and weak association with the planned date of quitting (H4b, β = 0.149 [95% CI 0.118, 0.179], *p* < 0.0001).Relevance had a positive moderate association with hypothetical PD (H2b, β = 0.577 [95% CI 0.521, 0.634], *p* < 0.0001), and in turn, had a positive and moderate association with the importance of quitting (H5a, β = 0.309 [95% CI 0.276, 0.343], *p* < 0.0001) and perceived importance of quitting (H5c, β = 0.383 [95% CI 0.342, 0.425], *p* < 0.0001) as well as had a negative and moderate association with the planned date of quitting (H5b, β = − 0.307 [95% CI − 0.341, − 0.274], *p* < 0.0001). Furthermore, (H1c) hypothetical PD had a direct positive and moderate association with intention to quit (β = 0.535 [95% CI 0.509, 0.561], *p* < 0.0001) and its perceived importance (β = 0.663 [95% CI 0.631, 0.695], *p* < 0.0001) and a direct negative association with its planned date (β = − 0.532 [95% CI − 0.559, − 0.505], *p* < 0.0001). The higher the perceived likelihood of a negative cost of smoking was, the higher the intention to quit and its importance were, and the closer in time the planned date was. The model explained 28.7% of the variance of intention to quit smoking, 28.3% of the planned time of cessation, and 44.0% of reported importance of quitting smoking.

**Table 4:** Mediation analyses including direct and indirect effects for A: Inaction-cost Model; B: Inaction-gain Model; and C: Action-gain Model.

A		
	*β*	95% CI
	Direct effects	
Temporal PD → hypothetical PD (H1)	− 0.280 (*p* < 0.001)	[− 0.336, − 0.224]
Relevance → hypothetical PD (H2b)	0.577 (*p* < 0.001)	[0.521, 0.634]
Temporal PD → relevance (H2a)	− 0.759 (*p* < 0.001)	[− 0.779, − 0.738]
hypothetical PD → intention to quit (H3a)	0.535 (*p* < 0.001)	[0.509, 0.561]
hypothetical PD → planned date of quitting (H3b)	− 0.532 (*p* < 0.001)	[− 0.559, − 0.505]
hypothetical PD → importance of quitting (H3c)	0.663 (*p* < 0.001)	[0.631, 0.695]
	Indirect effects	
Temporal PD → hypothetical PD → intention to quit (H4a)	− 0.150 (*p* < 0.001)	[− 0.180, − 0.119]
Temporal PD → hypothetical PD → planned date of quit (H4c)	0.149 (*p* < 0.001)	[0.118, 0.179]
Temporal PD → hypothetical PD → importance of quit (H4b)	− 0.186 (*p* < 0.001)	[− 0.224, − 0.148]
Relevance → hypothetical PD → intention to quit (H5a)	0.309 (*p* < 0.001)	[0.276, 0.343]
Relevance → hypothetical PD → planned date of quit (H5c)	− 0.307 (*p* < 0.001)	[− 0.341, − 0.274]
Relevance → hypothetical PD → importance of quit (H5b)	0.383 (*p* < 0.001)	[0.342, 0.425]

B		
	*β*	95% CI
	Direct effects	
Temporal PD → hypothetical PD (H1)	− 0.273 (*p* < 0.001)	[− 0.359, − 0.187]
Relevance → hypothetical PD (H2b)	0.583 (*p* < 0.001)	[0.493, 0.672]
Temporal PD → relevance (H2a)	− 0.751 (*p* < 0.001)	[− 0.781, − 0.722]
hypothetical PD → intention to quit (H3a)	− 0.143 (*p* < 0.001)	[− 0.169, − 0.118]
hypothetical PD → planned date of quitting (H3b)	0.118 (*p* < 0.001)	[0.093, 0.144]
hypothetical PD → importance of quitting (H3c)	− 0.128 (*p* < 0.001)	[− 0.154, − 0.102]
	Indirect effects	
Temporal PD → hypothetical PD → intention to quit (H4a)	0.039 (*p* < 0.001)	[0.025, 0.053]
Temporal PD → hypothetical PD → planned date of quit (H4c)	− 0.032 (*p* < 0.001)	[− 0.045, − 0.020]
Temporal PD → hypothetical PD → importance of quit (H4b)	0.035 (*p* < 0.001)	[0.022, 0.048]
Relevance → hypothetical PD → intention to quit (H5a)	− 0.084 (*p* < 0.001)	[− 0.103, − 0.064]
Relevance → hypothetical PD → planned date of quit (H5c)	0.069 (*p* < 0.001)	[0.051, 0.087]
Relevance → hypothetical PD → importance of quit (H5b)	− 0.075 (*p* < 0.001)	[− 0.094, − 0.056]

C		
	*β*	95% CI
	Direct effects	
Temporal PD → hypothetical PD (H1)	− 0.391 (*p* < 0.001)	[− 0.418, − 0.363]
Relevance → hypothetical PD (H2b)	0.526 (*p* < 0.001)	[0.501, 0.551]
Temporal PD → relevance (H2a)	− 0.451 (*p* < 0.001)	[− 0.471, − 0.431]
hypothetical PD → intention to quit (H3a)	0.565 (*p* < 0.001)	[0.537, 0.593]
hypothetical PD → planned date of quitting (H3b)	− 0.583 (*p* < 0.001)	[− 0.613, − 0.554]
hypothetical PD → importance of quitting (H3c)	0.687 (*p* < 0.001)	[0.652, 0.722]
	Indirect effects	
Temporal PD → hypothetical PD → intention to quit (H4a)	− 0.221 (*p* < 0.001)	[− 0.240, − 0.202]
Temporal PD → hypothetical PD → planned date of quit (H4c)	0.228 (*p* < 0.001)	[0.209, 0.247]
Temporal PD → hypothetical PD → importance of quit (H4b)	− 0.269 (*p* < 0.001)	[− 0.291, − 0.246]
Relevance → hypothetical PD → intention to quit (H5a)	0.297 (*p* < 0.001)	[0.277, 0.317]
Relevance → hypothetical PD → planned date of quit (H5c)	− 0.307 (*p* < 0.001)	[− 0.328, − 0.286]
Relevance → hypothetical PD → importance of quit (H5b)	0.361 (*p* < 0.001)	[0.337, 0.386]

Bootstrapped confidence intervals were based on 5,000 replications and were estimated with diagonally weighted least squares. β = standardized regression weights, 95% CI bias-corrected bootstrapped confidence intervals.

The Inaction-gain Model showed an acceptable fit to the data; CFI of 0.934, TLI of 0.922 and RMSEA of 0.091 [90% CI 0.089–0.095]. The analyses showed (see Table 4B and Fig. 4B) that temporal PDs had a strong positive association with personal relevance (H1a, β = − 0.751 [95% CI − 0.781, − 0.722], *p* < 0.0001) and a moderate negative association with hypothetical PDs (H1b, β = − 0. 273 [95% CI − 0.359, − 0.187], *p* < 0.0001). The more distant the gains of smoking were perceived in the future, the lower the personal relevance and the hypothetical distance were. Furthermore, (H1c) hypothetical PD had weak negative association with intention to quit (β = − 0.143 [95% CI − 0.169, − 0.118], *p* < 0.0001) and its perceived importance (β = − 0.128 [95% CI − 0.154, − 0.102], *p* < 0.0001) as well as a weak positive association with planned date (β = 0.118 [95% CI 0.093, 0.144], *p* < 0.0001). The higher the perceived likelihood of a positive consequence of smoking was, the lower the intention to quit and its importance were, and the later in time the planned date was. The model explained 2.1% of the variance of intention to quit smoking, 1.4% of the planned time of cessation, and 1.6% of reported importance of quitting smoking.

The Action-gain Model showed an excellent fit to the data; CFI of 0.974, TLI of 0.970 and RMSEA of 0.052 [90% CI 0.049–0.056]. The analyses showed (see Table 4C and Fig. 4C) that temporal PDs had a moderate negative association with personal relevance (H1a, β = − 0.451 [95% CI − 0.471, − 0.431], *p* < 0.0001) and hypothetical PDs (H1b, β = − 0.391 [95% CI − 0.418, − 0.363], *p* < 0.0001). The more distant the costs of smoking were perceived in the future, the lower the personal relevance and the hypothetical distance was. Further, (H1c) hypothetical PD had a moderate positive association with intention to quit (β = 0.565 [95% CI 0.537, 0.593], *p* < 0.0001) and its perceived importance (β = 0.687 [95% CI 0.652, 0.722], *p* < 0.0001) as well as a moderate negative association with its planned date (β = − 0.583 [95% CI − 0.613, − 0.554], *p* < 0.0001). The higher the likelihood of a negative cost of smoking was perceived, that higher the intention to quit and its importance were, and the closer in time the planned date was. Further, temporal PDs mediated by hypothetical PDs had a negative and weak association with intention to quitting (H4a, β = − 0.221 [95% CI − 0.240, − 0.202], *p* < 0.0001),perceived importance of quitting (H4c, β = − 0.269 [95% CI − 0.291, − 0.246], *p* < 0.0001), and had a positive and weak association with the planned date of quitting (H4b, β = 0.228 [95% CI 0.209, 0.247], *p* < 0.0001). The model explained 31.4% of the variance of intention to quit smoking, 34.1% of the planned time of cessation, and 46.5% of reported importance of quitting smoking.

Since the Action-cost Model did not fit well to the data (CFI of 0.822, TLI of 0.794 and RMSEA of 0.131 [90% CI 0.134–0.128]) and explained only a small proportion of the variance of intention to quit smoking (5.2%), planned time of cessation (3.6%) and reported importance of quitting smoking (3.0%), associated results are not reported in the present study.

Model Rational showed acceptable fit to the data; CFI of 0.975, TLI of 0.926 and RMSEA of 0.089 [90% CI: 0.065, 0.115]. The difference of PD and relevance weighted positive and negative consequences of action showed a positive association with intention to quit smoking (β = 0.259 [95% CI 0.079, 0.440], *p* < 0.0001), and a negative association with the planned time of quit smoking (β = − 0.227 [95% CI − 0.410, − 0.043], *p* < 0.0001). The higher the summed probability of the weighted positive consequences of quitting smoking relative to the negative consequences was, the higher the willingness to quit smoke and its planned time closer in the future was. The difference of the PD and relevance weighted positive and negative consequences of inaction showed a negative association with intention to quit smoking (β = − 0.400 [95% CI − 0.522, − 0.279], *p* < 0.0001), and a positive association with the planned time of quit smoking (β = 0.418 [95% CI 0.297, 0.539], *p* < 0.0001). The higher the overall probability of subjectively weighted positive consequences of continuing smoking compared to negative consequences was, the lower the intention to quit and the further in the future the planned date were. The model explained 22.7% of the variance of intention to quit smoking, and 22.6% of the planned time of cessation.

Model Rational-control showed an acceptable fit to the data; CFI of 0.983, TLI of 0.950 and RMSEA of 0.062 [90% CI 0.034, 0.095]. The analyses showed that the difference of relevance weighted positive and negative consequences of smoking had a negative association with intention to quit smoking (β = − 0.120 [95% CI − 0.170, − 0.069], *p* < 0.0001), and a positive association with the planned time of quit smoking (β = 0.130 [95% CI 0.080, 0.181], *p* < 0.0001). The higher the summed probability of the subjectively weighted positive consequences of smoking relative to the negative consequences was, the lower the willingness to quit smoke and its planned time is more distant in the future were. The model explained 1.4% of the variance of intention to quit smoking, and 1.7% of the planned time of cessation.

## Discussion

Smoking is a serious threat to smokers' and their surroundings’ health, but personal behaviour might not reflect this. To better understand this discrepancy, we investigated psychological distances (PDs) towards smoking-related consequences and their relationship with smoking behaviour. The concept of PDs was developed by Liberman and Trope^6^, according to which the behavioural modification effect of future events depends on the perceived temporal (how close in time) and hypothetical (how likely it is going to happen) distances of that event. Adopting this concept, we examined how closely smokers perceive the costs and gains of smoking, and the costs and gains of stopping smoking and their relation to antismoking intention.

First, the present results are in line with previous studies (e.g., climate:^9,12,48,49^, smoking:^7^), suggesting that PDs are associated with protective behaviour. No prior study has investigated PDs of costs and gains of smoking and cessation to explain addictive behaviour among smokers. Consistent with our hypotheses, negative consequences of smoking and positive consequences of quitting perceived closer in the future were associated with higher personal relevance and higher perceived likelihood, which, in turn, was associated with a stronger intention to quit, its planned date was closer and the attributed importance of quitting was higher. These findings are consistent with the temporal discounting perspective, according to which there is a general tendency for people (especially for smokers^19^) to discount the value and significance of events and outcomes that occur in the future^50^.

The current results indicate that research on PDs should not be limited to health consequences but to other costs and benefits—such as stress-coping, recreation or psychological harms, desired lifestyle, (see^33^ or^34^)— that can be interpreted within the framework of Construal Level Theory. As Kim and Kim^7^ pointed out, cognitive frames—which involve PDs—are relatively stable constructs and are generally difficult to change using external input^51^. Thus, successful communication should not try to alter already existing PDs, but rather activate pre-existing ones^7^ with near-future consequences to increase the personal importance of a particular action. Considering that health threat is generally perceived in the distant future, while other losses (e.g. psychological wellbeing, desired lifestyle, fitness) are perceived to have an immediate negative effect (see Fig. 1 and Table 1), their application in antismoking communication might be beneficial.

Today, antismoking communication is largely dominated by threatening messages that depict health consequences of smoking^52–54^, even though their efficacy in evoking motivation to quit smoking remains inconclusive. The current results partly support the use of such warning labels, as PDs of inaction-costs were related to antismoking-behaviour. However, for many smokers, fear-eliciting (e.g., loss-framed) messages trigger stress response and in turn defensive avoidance^55^, which prevents cognitive processing of the message^56^. According to a meta-analysis, gain-framed messages might relate more strongly to prevention behaviours (e.g., smoking cessation) than loss framed messages^57^, in line with the prediction of Rothman and Salovey’s Framing theory^58^. Also, overfamiliar antismoking message frames—like health inaction-cost—may activate greater message fatigue and in turn lower intentions to quit smoking^59^. Considering the current results, that action-gain PDs are also closely linked to smoking cessation intention, the communication benefits associated with smoking cessation potentially have some advantages over the health inaction-cost frames. Initially, gain-framed messages do not elicit a stress response^55^, increasing the probability of message processing. Furthermore, it is worth noting the more immediate temporal psychological distances associated with taking action as opposed to inaction, particularly when considering health-related consequences. Median perceived temporal distance of health-related consequences of continuing smoking was in the 11– 20 years range, while in the action scenario median temporal distance of improved health was in the 3–6 months range (see Fig. 3). As proximal (vs. distal) time frame of smoking health-consequences led to greater perceived message relevance^60^, messages communicating health consequences of action-gain (gain-framed messages) may enhance receptivity. Within the Construal Level Theory framework^5,6^, individuals tend to perceive long-term consequences in a more abstract manner. This often leads to the formulation of similarly abstract and wide-ranging solutions, such as the thought, “I need to quit smoking.” These abstract solutions may not prompt immediate action. Conversely, short-term consequences are typically viewed in a more concrete light, leading individuals to take more immediate and focused steps toward achieving their goal. For example, they might think, “To quit smoking, I need to learn techniques that can assist me.” Such techniques might have particular importance, considering that the smoking related rituals and cessation-related craving are both rated as high personal relevance and are both perceived close in time. Furthermore, the median perceived psychological distance of cessation-related positive consequences (other than health) also fell within the 3–6-month range. This is significantly closer compared to the perceived distance of adverse health consequences of smoking (frequently used in health communication), which typically range from 11 to 20 years. These observations lead to the conclusion that anti-smoking health communication could be potentially enhanced by incorporating (1) action-gain framed messages and (2) a wider array of consequences, encompassing factors like improved psychological well-being, lifestyle, fitness, and appearance. In order to validate these hypotheses, it would be advantageous to conduct future studies examining the effectiveness of such health messages. Although, Nan et al.^16^ did not find a significant effect of message framing (gain vs. loss), and temporal framing (present-oriented vs. future-oriented) on the effectiveness of cigarette warning labels, their results can only be generalised with caution, as the sample only included non-smokers and only messages focusing on health consequences were tested.

Another question of the study was whether smoking is a rational behaviour based on the balance of costs and gains of action and inaction weighted with the corresponding PDs. According to Becker and Murphy^20^, if the expected gain outweighs the expected costs of quitting, a rational smoker will choose to quit. The results indicate that the intention of quitting smoking is related to the PD weighted gain-cost balance, by which smoking can be considered partly rational. Psychological distances proved to be an important factor in the cognitive evaluation process of smoking behaviour. The model with PD weighted consequences (Model Rational) explained 22% of the variance in the intention of antismoking behaviour, while the model without PDs (Model Rational-control) explained less than 2%. It is important to note, however, that it does not mean that smokers are “happy addicts”^61^, choosing their addiction after careful consideration. A key factor in developing an addiction is the underestimation of the likelihood of developing an addiction, resulting in regret of past consumption decisions^62^. The current results are consistent with this, as 71.2% of the sample wishing they had never started smoking. That is, although the evaluation process might be partly rational at the given moment (when an addiction has already developed), it does not necessary mean that smoking per se is rational. The current results indicate that the intention to quit largely depends on the evaluation of negative consequences of smoking and harm reduction (see the Inaction-cost and Action-gain Models), while positive consequences of smoking are almost irrelevant (see the Inaction- gain and Action-cost Models). This may not be considered rational for a pleasure-seeking behaviour, although it is in line with a number of studies showing that addictions are not usually driven by positive emotions^63^. The apparent contradiction between the rational and irrational aspects of smoking may be due to the fact that the stages of smoking may differ in terms of rationality^64^. Entry into smoking is irrational (e.g., affected by peer-group pressure, sentiments, fashion)^64^, but a smoker’s rationality plays a critical role in their decision to quit^65^. Since the role of subjective (dis)beliefs play an important role in the repeated use of addictive substances^61^, including temporal and probabilistic properties of harm following the behaviour^65^, the study of PDs may lead to a better understanding of the rationality behind the decision process. Defining and understanding rational and irrational states and their associated determinant decision processes can be useful for targeting health communication, as rational thought processes (as opposed to irrational ones) are more tentative, flexible, and consistent with reality and more supportive of one’s long-term goals^66^.

### Limitations and future directions

Although the study was conducted in a relatively large sample of smokers, some limitations need to be addressed. We used cross-sectional, self-reported data from a self-selected sample that may introduce biases (e.g., recall bias). Although mediation analyses conducted on cross-sectional data provide valuable preliminary insights into potential causal mechanisms, caution is warranted in interpreting these findings. Cross-sectional mediation analyses are susceptible to issues of confounding and reverse causality^67^, thus longitudinal studies are needed to establish temporal relationships and corroborate the proposed mediation pathways. Further, the absence of preregistration should be considered as a limitation, which may introduce the possibility of hindsight bias and could impact the robustness of the findings. Furthermore, minority groups had no option to specify their gender in the survey, a factor that might reduce their likelihood of participation and introduce potential biases in the sample. .Another important limitation of the study was that only quitting intentions were assessed, which is often different from actual quitting attempts and especially from successful quitting attempts^68^. In order to identify consequences relevant in practice, the message effectiveness of different consequences should be tested in the future, preferably in a longitudinal design. Although the possible practical implications of health communication have been discussed, given the public health importance of the topic, more empirical work should be conducted.

## Conclusions

Our findings provide useful insights into how smokers perceive various consequences of smoking and quitting, and their relation to antismoking intention. The results fit well with the literature on framing health messages and provides a detailed baseline of the perceived temporal and hypothetical properties of smoking consequences. Our results suggest that diversifying the themes of anti-smoking communication to include action-gain warning labels that are not limited to health consequences has the potential to increase effectiveness.

### Supplementary Information

Supplementary Information.

## Data Availability

Data is available at https://zenodo.org/records/10400781?token=eyJhbGciOiJIUzUxMiJ9.eyJpZCI6ImFiYWExOWU4LTc5NjYtNGUzMi1iNDE3LWE3NWQxMjlmOGZmNSIsImRhdGEiOnt9LCJyYW5kb20iOiJiYzkwZDliNDliN2RhMThmYzhiYTFlNWIwYzYyNTZlNSJ9.iARBKonEIUaNP1pzRONUd0M7srcwmP6IK_l-goWc_JlhbMbgcPHqY9oSVIL2N0EJu8dQCBh3SHV-k8Gc_PugVQ.

## Code availability

All the data on which the conclusions are based, along with the associated analysis scripts, accessible on Zenodo.com via the following link: https://zenodo.org/records/10400781?token=eyJhbGciOiJIUzUxMiJ9.eyJpZCI6ImFiYWExOWU4LTc5NjYtNGUzMi1iNDE3LWE3NWQxMjlmOGZmNSIsImRhdGEiOnt9LCJyYW5kb20iOiJiYzkwZDliNDliN2RhMThmYzhiYTFlNWIwYzYyNTZlNSJ9.iARBKonEIUaNP1pzRONUd0M7srcwmP6IK_l-goWc_JlhbMbgcPHqY9oSVIL2N0EJu8dQCBh3SHV-k8Gc_PugVQ.
