# Supplementary material for: Examining the impact of perceived psychological distances of quitting and continuing tobacco smoking on antismoking intention: a cross-sectional study
Source: Sci Rep. 2023 Dec 27;13:22993. doi: 10.1038/s41598-023-50440-6 (PMC10752891; doi:10.1038/s41598-023-50440-6)
Supplement: Supplementary file 1 — Supplementary Information. [file 41598_2023_50440_MOESM1_ESM.docx]

Appendix 1. Items used to assess smoking consequences of action and inaction.

Action

Temporal: Imagine you made the decision to stop smoking. Do you think the following would happen to you sooner or later?

Hypothetical: Once you've made the decision to stop smoking and had your last cigarette, how long do you think it would take for these changes to take effect?

1. I will miss smoking in certain situations (e.g. with coffee or alcohol, after a meal or during a break at work). (cost-ritual)

2. I will have more energy and be physically fitter. (gain-fitness)

3. I will feel less comfortable in others’ company. (cost-social facilitation)

4. I will look better (e.g. nicer teeth and skin, smell better). (gain-appearance)

5. My health will improve. (gain-health)

6. It will be harder to keep my weight at the right level. (cost-weight control)

7. I will be more irritable and find it harder to handle stress. (cost-stress)

8. People around me will be proud of me. (gain-social)

9. I will be able to lead a more balanced lifestyle. (gain-lifestyle)

10. I'll crave smoking. (cost-craving)

11. My financial situation will improve significantly (e.g. due to the price of tobacco, dentist). (gain-financial status)

12. I will feel freer and better overall. (gain-psychological wellbeing)

13. My life becomes emptier and more boring (cost-enhancement)

14. I will have reduced performance at work or school (e.g., due to distraction) (cost-performance)

Inaction

Hypothetical: Imagine you decide not to quit smoking! What do you think would happen to you sooner or later?

Temporal: Imagine that you decide not to quit smoking: approximately how long would it take for you to experience the following?

1. My life will be more fulfilling with smoking rituals (e.g. with coffee or alcohol, after a meal or during a break at work) (gain-ritual)

2. I will have less energy and be less physically fit. (cost-fitness)

3. I will have more social interactions (e.g. smoking at work). (gain-social facilitation)

4. My appearance will deteriorate (e.g. worse teeth, wrinkled skin, cigarette smell) (cost-appearance)

5. I will develop a serious illness as a result of smoking. (cost-health)

6. It will be easier to keep my weight at the right level. (gain-weight control)

7. I will find it easier to handle difficult situations. (e.g. anxiety, stress) (gain-stress)

8. People around me will be bothered by my smoking. (cost-social)

9. It will bother me that an addiction is present in my life. (cost-lifestyle)

10. My financial situation will deteriorate significantly (e.g., price of tobacco, dentist). (cost-financial status)

11. Overall, my life will be more exciting. (gain-enhancement)

12. I'll feel guilty, I'll feel worse about myself. (cost-psychological wellbeing)

13. I will have better performance at work or school (e.g., due to better focus) (gain-performance)

**Appendix 2.** **Demographic and socioeconomic status**

1. Sex
   1. Female
   2. Male
   3. Prefer not to answer
2. Age
   1. Answer can be indicated on a slide from 18 to 100
3. Education (highest degree completed)
   1. Primary education
   2. Vocational degree
   3. High-school degree
   4. Bachelor’s degree
   5. Master’s degree
   6. Ph.D. degree
4. Relationship status
   1. Single
   2. In a relationship
   3. In civil partnership
   4. Married
   5. Widowed
   6. Separated
   7. Other

**Appendix 3. Personal relevance of smoking consequences.** Participants were asked to indicate the importance of each consequence using a five-point Likert scale, ranging from 1 – not at all to 5 – very important. +: positive consequence; -: negative consequence

- 1. Helps me connect with others (+)
  2. Unhealthy (-)
  3. Rituals related to smoking (+)
  4. Costs a lot of money (-)
  5. Helps me to concentrate (+)
  6. Doesn't fit in with the lifestyle I want (-)
  7. Makes me feel guilty (-)
  8. Disturbs my environment (-)
  9. It brings greater fulfillment to my life (+)
  10. Helps me manage stress (+)
  11. It makes me less fit (-)
  12. It negatively affects my appearance (-)
  13. It aids in overcoming cravings (+)
  14. Helps me keep my weight at the right level (+)
